# *Midnolin* is a confirmed genetic risk factor for Parkinson’s disease

**DOI:** 10.1002/acn3.50914

**Published:** 2019-10-06

**Authors:** Yutaro Obara, Hidenori Sato, Takahiro Nakayama, Takeo Kato, Kuniaki Ishii

**Affiliations:** ^1^ Department of Pharmacology Yamagata University School of Medicine Yamagata Japan; ^2^ Genome Informatics Unit Institution for Promotion of Medical Science Research Yamagata University School of Medicine Yamagata Japan; ^3^ Research Institute of Bio‐system informatics Tohoku Chemical Co., LTD Morioka Japan; ^4^ Yamagata City Institute of Public Health Yamagata Japan

## Abstract

**Objective:**

Genetic analysis of patients with familial Parkinson’s disease (PD) identified many causative genes. However, the majority of PD cases are sporadic, and the mechanisms of onset still remain unclear. Previously, we found that *Midnolin* (*MIDN*) is associated with PD in a Yamagata (Japan) cohort study and that MIDN regulates neurite outgrowth and Parkin expression in neuronal cells. In the present study, we aimed to replicate the genetic association between *MIDN* and PD in a large British population cohort.

**Methods:**

In this replication study, we analyzed the copy number variations and single‐nucleotide polymorphisms of the *MIDN* gene in a large British population on a case–control genome‐wide association study dataset including 2,860 controls and 2,168 PD patients.

**Results:**

There was significant copy number loss in the *MIDN* gene with an odds ratio of 4.35 (*P* < 2.2 × 10^−16^). Furthermore, there were many patients in both the British and Yamagata case groups who have a long spanning deletion. The odds ratio dramatically increased to 22.3 (*P* = 3.59 × 10^−15^) when a deletion spanning more than 50,000 bp was defined as the copy number loss. There were no significant differences between the controls and study cases for two relatively frequent single‐nucleotide polymorphisms (rs3746106 and rs3746107).

**Interpretation:**

We showed the strong genetic association of *MIDN* with PD development in a British population and in a Japanese population, suggesting *MIDN* is a confirmed and universal genetic risk factor for PD.

## Introduction

Parkinson’s disease (PD) is the second most common neurodegenerative disease and is characterized by motor impairments including rest tremor, akinesia, and rigidity. In general, dopaminergic neurons in the substantia nigra projecting to the striatum are degenerated, resulting in PD onset. Genetic analysis of patients with familial PD identified more than 20 causative genes with rare and highly penetrant mutations, including *SNCA*, *Parkin*, *PINK1,* and *LRRK2*.^1^, ^2^, ^3^ The pathophysiological roles of these genes have been extensively studied. In addition, although relatively common, genetic risk variants with modest effects have been demonstrated, including *GBA* and *INPP5F*.^1^, ^4^ However, the majority of PD cases are sporadic, and the mechanisms of onset still remain unclear.

In Britain, a large‐scale molecular epidemiological study related to sporadic PD (sPD) was performed by the Wellcome Trust Case Control Consortium 2 (WTCCC2) (http://www.wtccc.org.uk). Remarkable results have been reported from this study combined with or without multiple cohort studies. For example, six previously reported loci and five new loci were identified as genetic risks for PD.^5^ Furthermore, 8 of 9387 PD patients and none of the 13,863 controls had a large hemizygous deletion (>3 Mbp) at 22q11.2, and this deletion was more prevalent in patients with early‐onset PD than in those with late‐onset PD.^6^

In 2000, the *Midnolin* (*MIDN*) gene was discovered in embryonic stem cells.^7^ MIDN is abundantly expressed in embryonic midbrain and localizes in the nucleus and nucleolus. A ubiquitin‐like domain and nucleolar localization signal are included in the N‐terminal and C‐terminal regions of MIDN, respectively, and MIDN is assumed to be involved in neurogenesis regulation.^7^ Since its discovery, it has been reported that the ubiquitin‐like domain interacts with glucokinase and that MIDN overexpression inhibits insulin secretion from MIN6 cells.^8^ *MIDN* was also identified as a candidate gene implicated in female autism spectrum disorders.^9^

Previously, we have found that nerve growth factor (NGF) promotes neurite outgrowth and catecholamine biosynthesis in PC12 cells accompanied by activation of extracellular signal‐regulated kinases 1/2 and 5.^10^, ^11^, ^12^ Furthermore, we revealed that *MIDN* is upregulated by NGF through these kinases,^13^ suggesting MIDN has essential roles in neuronal development. Importantly, we discovered that there was *MIDN* copy number (CN) loss in 10.5% of sPD patients in Yamagata Prefecture, Japan, whereas there was no CN loss in the control group, indicating *MIDN* involvement in the pathogenesis of sPD.^13^ NGF‐induced neurite outgrowth and expression of the ubiquitin E3 ligase, Parkin, were largely suppressed in PC12 cells in which *MIDN* gene expression was silenced.^13^ Furthermore, it has been shown by transcriptome analysis that the mRNA expression of a large number of genes, including various PD causative genes, is regulated by MIDN, suggesting that MIDN is a transcription modulator, although no obvious DNA‐binding domain or transcription‐activating domain is encoded in the *MIDN* gene.^14^

From the molecular epidemiological point of view, it is necessary to replicate our results from the Yamagata cohort study in other populations. Therefore, we examined the possibility of genetic association between *MIDN* and PD in a British population cohort, using the WTCCC2 cohort data.

## Subjects and Methods

### Subjects

WTCCC2 cohort datasets (EGAD00000000022 for control, EGAD00000000057 for case) were officially obtained from the European Genome Archive (https://ega-archive.org). General population control samples are derived from the 1958 British Birth Cohort (58C) (also known as the National Child Development Study). 58C is a sample of sequential live births in the UK during 1 week in 1958.^15^ The approximate age at genotyping of the controls from 58C was 52 years old.^16^ Patients with idiopathic PD enrolled in this study were diagnosed on the basis of the UK Brain Bank Clinical Criteria for PD.^17^ Patients with an apparent family history have been excluded. The individual age of onset was not available, but it has been reported that the mean age of disease onset of 1439 samples from the same dataset was 65.8, with the youngest being 29 years and the oldest 105 years,^18^ which is very similar to the mean age of onset in the Yamagata population (64.7).^13^

### Analysis of CN variations (CNVs) and single‐nucleotide polymorphisms (SNPs)

Samples were genotyped using Illumina 1.2M and 660K Quad arrays for controls and study cases, respectively (Illumina, San Diego, CA, USA). For CNV analysis, the estimated CN was calculated by the Hidden Markov Model algorithm using the Unix‐based PennCNV program^19^ from both the B‐allele frequency data and the signal intensity data (LogR Ratio) normalized by standard deviation (SD) of intra‐ and interchips, which were designed to target the frequent SNP regions and the CN regions. In this analysis, we included CNVs identified with at least three markers. Samples were excluded if gender information was not available or their inferred gender was discordant with the recorded gender, determined by the B‐allele frequency of chromosome X (seven samples of the controls and 261 samples of the study cases were finally excluded). The statistical significance of differences in CNVs and SNPs were analyzed with Fisher’s exact test.

The present study was performed in accordance with a protocol approved by the Ethics Committee of Yamagata University (approval no. 90).

## Results

**Figure 1:**
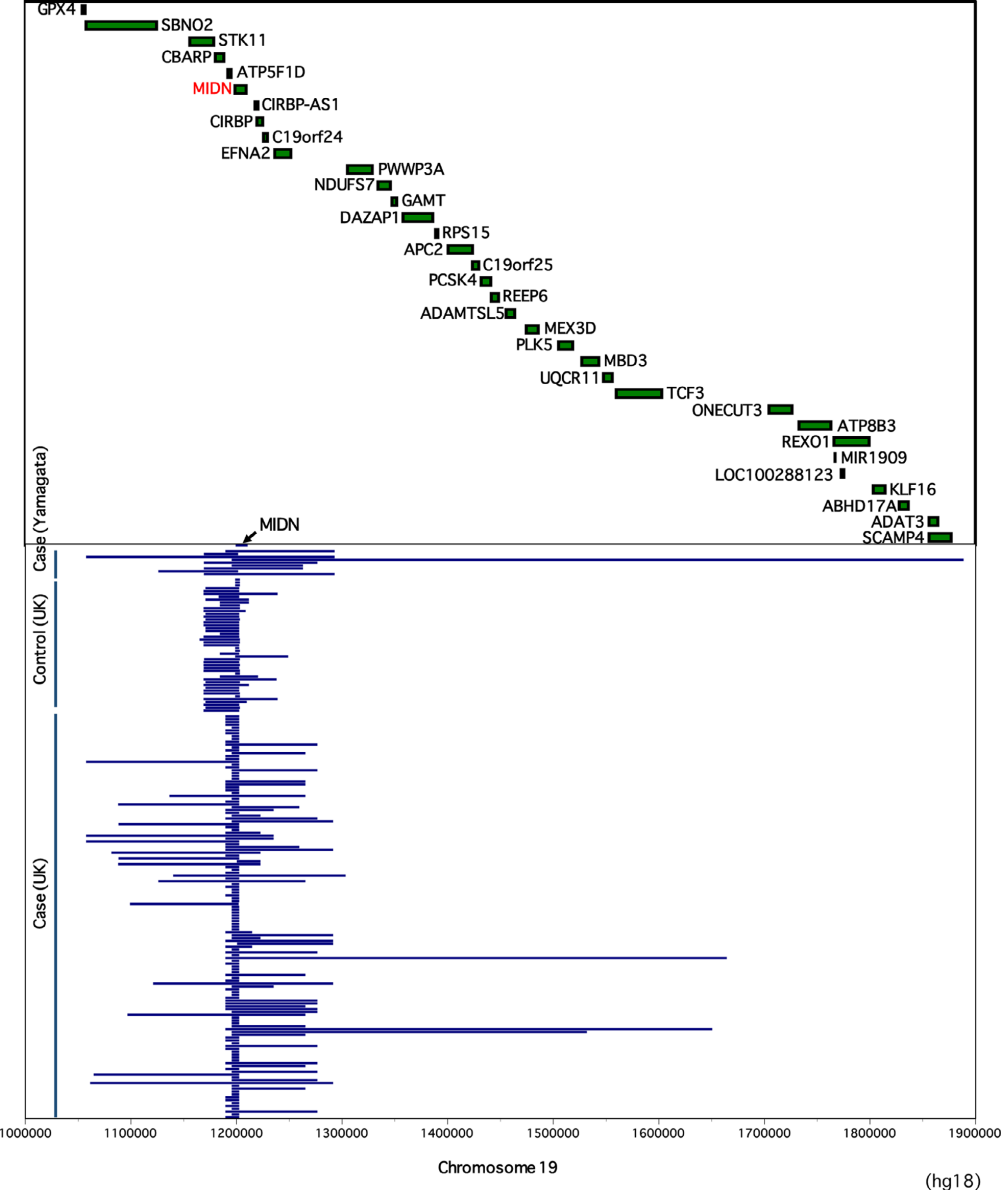
Genomic location of *MIDN* deletions found in 47 controls and 151 PD patients. The 151 patients include 9 and 142 patients from the Yamagata cohort and the UK WTCCC2 cohort, respectively.

We analyzed the CNVs and SNPs of the *MIDN* gene in 2860 controls and 2168 PD patients. Under our criteria for CNV detection, we found that 1.64% of the controls and 6.55% of the patients had CN loss (CN = 1; Table1). The genomic location of *MIDN* deletions is shown in Figure 1. The *MIDN* deletion rate was 3.99‐fold higher in study cases than in controls. In the Yamagata cohort study, 10.5% of the PD patients had *MIDN* CN loss. This percentage is smaller in the British population cohort, but 6.55% is still a high rate. The odds ratio was 4.35 in this study. In both control and case groups, there were people with entire or segmental multiplication of *MIDN* gene (CN = 3 or 4) (Table S1). It is assumed that only transcripts of multiplication of the entire region of the *MIDN* gene can be functional, however, there is no evidence that suggests that this multiplication is associated with PD. Conversely, transcripts of segmental multiplication of the *MIDN* gene may interfere with normal MIDN functions if their transcripts are expressed in frame. Alternatively, the *MIDN* gene may be disrupted if the segmental multiplication is introduced within the *MIDN* gene, which is equivalent to the CN decrease.

**Table 1:** *MIDN* CNV analysis of PD patients and controls.

	Total	CN = 2 (normal)	CN = 1 (loss)	CN = 3 or 4 (gain)
Control	2860 M1469, F1391	2804 M1445, F1359	47 (1.64%) M19, F28	9 M5, F4
Case	2168 M1359, F809	1947 M1211, F736	142 (6.55%) M91, F51	79 M57, F22

The *MIDN* gene CNVs in 2168 PD patients and 2860 controls were analyzed as described in the Methods. There was significant CN loss between study cases and controls with an odds ratio (OR) of 4.35 (*P* < 2.2 × 10^−16^, Fisher’s exact test compared between CN = 2 and CN = 1). M, male; F, female.

As shown in Figure 1, there were many patients in both the British and Yamagata case groups who have a long spanning deletion. Hence, *MIDN* deletion was defined as a region including a deletion of more than a 50,000 bp. We further analyzed the data with this definition (Table 2). We found that 0.105% of the controls and 2.21% of the patients had CN loss (CN = 1), and the *MIDN* deletion rate was 21.1‐fold higher in the study cases than in the controls. The odds ratio dramatically increased to 22.3 under this definition.

**Table 2:** Long spanning deletions of *MIDN* gene (>50,000 bp) in PD patients and controls

	CN = 2 (normal)	CN = 1 (loss)
Control	2848 M1462, F1386	3 (0.105%) M2, F1
Case	2041 M1269, F772	48 (2.21%) M33, F15

*MIDN* CN loss was defined as a deletion region including more than 50,000 bp. There was significant CN loss between study cases and controls with an odds ratio (OR) of 22.3 (*P* = 3.59 × 10^‐15^, Fisher’s exact test compared between CN = 2 and CN = 1). M, male; F, female.

We also analyzed two relatively frequent SNPs (rs3746106 and rs3746107) in the *MIDN* gene (Table 3). However, there were no significant differences between the controls and study cases (*P* = 0.309 for rs3746106, and *P* = 1 for rs3746107), as there was no significant difference in rs3746106 in the previous Yamagata cohort study.

**Table 3:** Association results of two SNPs in the *MIDN* gene.

(hg18)				
rs3746106	19:1200859, C> A		5'‐UTR	
	CC	AC	AA	N/A
Control (2860)	938 (32.8%)	1398 (48.9%)	515 (18.0%)	9 (0.315%)
Case (2168)	699 (32.2%)	1039 (47.9%)	423 (19.5%)	7 (0.323%)

rs3746107	19:1201147, C> T		34Ala> 34Ala synonymous	
	CC	CT	TT	N/A
Control (2860)	2697 (94.3%)	113 (3.95%)	0 (0.00%)	50 (1.75%)
Case (2168)	2076 (95.8%)	82 (3.78%)	2 (0.0923%)	8 (0.369%)

rs3746106 and rs3746107 correspond to the 5′‐UTR region and Ala34, respectively. There were no significant differences between controls and study cases (*P *= 0.309 for rs3746106, and *P *= 1 for rs3746107, Fisher's exact test).

## Discussion

In this study, the molecular epidemiological results from a Yamagata cohort study were replicated in a British population cohort, regardless of racial and regional differences. Hence, we propose that the CN loss of the *MIDN* gene is a universal genetic risk factor for PD.

In our Yamagata cohort study, *MIDN* deficiency was found in 10.5% of the PD patients, whereas none were found in the control group.^13^ This frequency is quite large compared with other genetic factors. In the current study, 6.55% of the patients had *MIDN* CN loss, although there were also *MIDN*‐deficient people in the control group. In the Yamagata cohort, all of the control participants were enrolled at age 70–71. In contrast, the British control participants were approximately 52 years old.^16^ Because the onset age was similar regardless of *MIDN* CN in the Yamagata cohort, those who have *MIDN* CN loss in the British control group may be relatively young for developing PD. A health examination for of the British control group will be performed in 2020 (at the age 62) and we need to follow‐up with a sequential study. Also, the PD patients with atypical symptoms were originally excluded from a Japanese cohort. Thus, it is speculated that the patients with *MIDN* loss found in a British population, may show typical symptoms including age of onset.

In the Yamagata cohort study, there were one male and eight females with decreased *MIDN* CN, and there was a tendency for *MIDN* gene loss to affect females.^13^ However, an obvious tendency in females was not observed in this British cohort study, suggesting a region‐specific difference. The reason for this discrepancy is currently unclear, but it may be related to the fact that in Japan there are more female PD patients than male patients, which is in contrast to the gender ratios in European countries and the United States.^20^

Furthermore, as a characteristic of *MIDN* deletion, the deletion length in the *MIDN* gene in the study cases was much larger than that in the controls in the British population (Fig. 1). In many cases, the deletion location spans multiple genes adjacent to the *MIDN* gene. When *MIDN* deletion was defined as a region including a deletion of more than a 50,000 bp, then the odds ratio was over 20 (Table 2). Furthermore, there was no person identified in the controls, when the deletion was defined as a region of more than 100,000 bp [0 patients (0.00%) in the controls and 21 patients (0.969%) in the study cases]. Because PD phenotypes were observed upon suppressing *MIDN* expression by genome‐editing or RNAi methods in neuronal cells,^13^ it is assumed that inhibition of MIDN functions is associated with PD onset or progress. However, we cannot exclude the possibility that another pathogenic gene(s) is involved in the development of PD in addition to *MIDN*. For example, *MIDN* deletion in the British population cohort (38 of 142 of study cases, and 4 of 47 of controls) spans *EFNA2*, encoding ephrin A2 protein (Fig. 1). Ephrin A2 is a ligand of ephrin A receptor tyrosine kinases (EphA); this bidirectional signaling regulates cell contact‐dependent axon guidance and synaptic plasticity in neuronal development. Because disruption of ephrin A/EphA signaling reduced dopaminergic innervation in the nigrostriatal system^21^ and ephrin A5 signaling is implicated in accurate projection of dopaminergic axons from the substantia nigra into the striatum,^22^ it is assumed that ephrin A2 similarly regulates the dopaminergic axon guidance, thus its loss of functions may also be associated with PD.

There were no individuals found in Yamagata and Britain with *MIDN* CN = 0, suggesting MIDN plays critical roles in neuronal development and its entire loss may be lethal. Therefore, it is necessary to clarify the detailed physiological role of MIDN in the future. To date, there are no PD patients with a family history of a genetic mutation or deletion in the *MIDN* gene. The patients’ family history is currently under examination in more detail. If patients with a family history will be found in the British or Yamagata cohort studies, it is possible that the deletion of the *MIDN* gene resulted from a *de novo* event that is passed on to their offspring, as these PD patients are originally defined as sporadic in both cohort studies. In addition, it is possible that *MIDN* is a novel causative gene for PD. Chromosome 19, where *MIDN* is encoded, has extremely high gene density and proportion of CNV sequences relative to other human chromosomes.^23^, ^24^ Chromosome 19 is rich in segmental duplications and tandemly clustered gene families, which may make it more prone to CNV^24^. CNVs frequently occur towards telomeres, and *MIDN* is located in this instable region (19p13.3), suggesting that *de novo* pathogenic CNV of *MIDN* may occur more frequently.

In this study, *MIDN* loss was associated with PD development in both a British and a Japanese population cohort, suggesting it is a confirmed and universal genetic risk factor for PD. Further studies on family history, functions in neuronal cells and physiological roles using knockout mice are necessary.

## Author Contributions

Y.O., T.K., and K.I. conceived and designed the data analysis. T.N. analyzed the cohort data, and H.S. supervised the analysis. Y.O. wrote the paper, and H.S., T.K. and K.I. revised it critically for important intellectual content. All authors reviewed the results and approved the final version of the manuscript.

## Conflicts of Interest

Takahiro Nakayama is an employee of Tohoku Chemical Co., LTD. (Hirosaki, Japan).

## Supporting information


**Table S1:** Stratification by entire and segmental multiplication of *MIDN* gene. Nine Controls and 79 patients with CN = 3 or 4 were stratified by entire or segmental multiplication of *MIDN* gene.
