# Supplementary material for: Midnolin is a confirmed genetic risk factor for Parkinson’s disease
Source: Ann Clin Transl Neurol. 2019 Oct 6;6(11):2205–11. doi: 10.1002/acn3.50914 (PMC6856597; doi:10.1002/acn3.50914)
Supplement: Supplementary file 1 — Table S1: Stratification by entire and segmental multiplication of MIDN gene. Nine Controls and 79 patients with CN = 3 or 4 were stratified by entire or segmental multiplication of MIDN gene. [file ACN3-6-2205-s001.docx]

|  | **CN=3** | | **CN=4** | |
| --- | --- | --- | --- | --- |
|  | **entire** | **segmental** | **entire** | **segmental** |
| **Control** | 2 (M0, F2) | 7 (M5, F2) | 0 | 0 |
| **Case** | 11 (M10, F1) | 46 (M31, F15) | 2 (M1, F1) | 20 (M15, F5) |

**Supplementary table 1: Stratification by entire and segmental multiplication of *MIDN* gene.** Nine Controls and 79 patients with CN = 3 or 4 were stratified by entire or segmental multiplication of *MIDN* gene. OR 7.92, *p* = 0.00271 (CN = 2 vs. CN = 3 (entire)); OR 9.12, *p* = 5.77 x 10^-14^ (CN = 2 vs. CN = 3 (all)); *p* = 0.168 (CN = 2 vs. CN = 4 (entire)); *p* = 3.24 x 10^-9^ (CN = 2 vs. CN = 4 (all)).
